# Fixation Time for Competing Beneficial Mutations and Their Genomic Footprint

**DOI:** 10.3390/biology14070775

**Published:** 2025-06-27

**Authors:** Wolfgang Stephan

**Affiliations:** Faculty of Biology, Ludwig-Maximilian University of Munich, D-82152 Planegg-Martinsried, Germany; w.stephan@lmu.de or stephan@bio.lmu.de; Tel.: +49-8841-6259920

**Keywords:** theoretical population genetics, nonnormalized allele frequencies, recurrent selective sweeps

## Abstract

For a highly beneficial mutation *A* at locus 1 spreading in a very large population, we have analyzed the scenario that at a closely linked locus 2 a second beneficial mutant *B* arises before *A* has fixed. Under the assumptions that the fitness of *B* is greater than that of *A* and that *A-* and *B*-carrying chromosomes can recombine at some rate r, recombinants *AB* may form and eventually fix. We present explicit formulas for the fixation time of *AB* under additive fitness of the mutants as a function of the frequency $X_{2}\left(0\right)\;$ of *A* at the time when *B* is introduced. Our analysis suggests that the effect of interference between the beneficial mutations is most pronounced for small values of $X_{2}\left(0\right)<0.1.$ Furthermore, we identify a threshold value for r, above which recombination speeds up fixation. Using published simulation data, we also describe the genomic footprint of competing beneficial mutations. At neutral sites between the two linked selected loci, an excess of intermediate-frequency variants may occur when interference is strong, i.e.,$\;X_{2}\left(0\right)$ small. Finally, we discuss under which circumstances this scenario may be encountered in real sequences from recombining genomic regions.

## Simple Summary

Knowing how fast natural selection can act on changes in the environment is fundamental for understanding evolution. The time it takes for a beneficial mutation to spread through a natural population has been investigated thoroughly in the history of population genetics and evolutionary biology. In this study the focus is on the interaction between beneficial mutations during their way through a population. The traditional view has been that beneficial mutations occur sequentially such that there is at most one of them on a chromosome on its way through a population at a time. The question, however, is what happens when they overlap. Do they compete with each other? Another notion is that in some circumstances they may recombine with each other such that their chance to spread through a population is increased. A mathematical model is presented to estimate the time until two overlapping beneficial mutations recombine and go to fixation; i.e., sweep together through an entire population. These dynamics are then linked to characteristic genomic patterns that may be observed in DNA sequencing studies.

## 1. Introduction

The speed of beneficial mutations on their way to fixation in natural populations is a fundamental topic in population genetics. Knowing how fast selection can act to change allele frequencies is essential for understanding evolution. The time for a beneficial allele to spread through a natural population was investigated early in the history of population genetics, using deterministic models [1]. Later, properties of the fixation time of individual advantageous mutations under the influence of positive directional selection and genetic drift in populations of finite size have been derived by several authors [2,3,4].

However, the spread of two or more beneficial mutations that arise and interact during their fixation process is much less investigated. For two interacting mutations Otto and Barton [5] have studied the case that the second mutation is less beneficial than the first one. In contrast, Cuthbertson et al. [6] and Bossert and Pfaffelhuber [7] considered the scenario that the second mutation is fitter than the first one. Furthermore, they assumed that there is a chance that the first and second mutants recombine such that the recombinant type has the highest fitness and eventually fixes. Here we will focus on this latter case.

We consider the mathematical analysis of the fixation time in conjunction with the theory of selective sweeps. Although beneficial mutations are a comparatively small fraction of all new mutations, some of them may reach fixation and are thus important in evolution. If the fitness effects of these beneficial mutations are sufficiently strong, they may cause selective sweeps, i.e., localized reductions of genetic variation along genomes [8]. Such localized patterns of reduced genetic variation have been convincingly described in a variety of organisms.

Detecting signatures of selective sweeps in genomes is a major goal of current population genetics, as it allows estimating the rate of beneficial mutations going to fixation and finding the genes involved in selection. The inference methods for detecting sweeps depend critically on assumptions on whether the beneficial mutations occur sequentially (such that there is at most one beneficial allele on a chromosome on the way to fixation at a time) or whether beneficial alleles overlap with each other. Models of recurrent selective sweeps traditionally assume that in chromosomal regions of normal recombination rates, at most one beneficial allele is on the way to fixation [9,10,11].

Here we follow the scenario proposed by Bossert and Pfaffelhuber [7]. Thus we assume that, while a highly beneficial mutation spreads in a natural population, a second beneficial mutation arises before the first one has fixed. Furthermore, we envision that the first mutation is less fit than the second one and that recombination may occur between the two mutations. Under these conditions a haplotype may be formed that is fitter than the two individual mutations and may therefore eventually fix. To model this process for a population of finite size, Bossert and Pfaffelhuber [7] used stochastic differential equations and calculated the fixation time of a recombinant haplotype under the assumption that it fixes. In contrast, we use a deterministic approach based on ordinary differential equations (ODEs). Thus, in our analysis an explicit assumption about the fixation of a recombinant is not necessary.

We begin by formulating the differential equations for the basic allele frequency changes. Then we introduce nonnormalized variables (that are proportional to the allele frequencies) to find approximate solutions of this system of ODEs. Subsequently, we provide explicit formulas for the fixation time of the recombinant type. Finally, we apply our results to simulation data by Chevin et al. [12] to describe patterns of selective sweeps in the genome caused by the joint fixation of two mutations due to selection and recombination.

## 2. Model

We consider a two-locus model, with alleles *A* and *a* at locus 1 and *B* and *b* at locus 2, respectively. The upper case letters denote beneficial alleles with selection coefficient $s_{2}$ at locus 1 and $s_{3}$ at locus 2, whereas the alleles with lower case letters are assumed to be neutral (wildtype). This model has four haplotypes *AB*, *Ab*, *aB*, and *ab*, with frequencies given by the variables $X_{1},\;X_{2},\;X_{3\;},\;$and $X_{4}$ (which add up to 1). Assuming additive selection, their relative fitnesses are $1+s_{1}$, $1+s_{2}$, $1+s_{3\;}$and 1, respectively, where $s_{1}=s_{2}+s_{3}$. Recombination between locus 1 and locus 2 occurs at rate *r*. Since we are interested in closely linked loci, we assume that $r\ll s_{i}\ll 1$ for *i* = 2, 3. In our deterministic setting (without genetic drift) the ODEs for the time change of the variables *X_i_* are obtained by adding the change due to selection and the change due to recombination ([13], chapt. 2):

(1) $$\begin{array}{c} \frac{dX_{1}}{dt}=X_{1}\left(s_{1}-\sum_{j=1}^{3}s_{j}X_{j}\right)-r\left(X_{1}X_{4}-X_{2}X_{3}\right)\;,\\ \frac{dX_{2}}{dt}=X_{2}\left(s_{2}-\sum_{j=1}^{3}s_{j}X_{j}\right)+r\left(X_{1}X_{4}-X_{2}X_{3}\right),\\ \frac{dX_{3}}{dt}=X_{3}\left(s_{3}-\sum_{j=1}^{3}s_{j}X_{j}\right)+r\left(X_{1}X_{4}-X_{2}X_{3}\right),\\ \frac{dX_{4}}{dt}=X_{4}\left(-\sum_{j=1}^{3}s_{j}X_{j}\right)-r\left(X_{1}X_{4}-X_{2}X_{3}\right), \end{array}$$

where t measures time in generations.

For r>0, which we assume throughout this paper, an exact solution of this system of ODEs is not known. An approximate solution can be obtained using nonnormalized variables *Y_i_* (*i* = 1, …, 4) (see [14] for mutation-selection and [15] for recombination-selection equations). These are related to the original variables of Equation (1) as:

(2) $$X_{i}=\frac{Y_{i}}{\sum_{j=1}^{4}Y_{j}}\;.$$

Using (2) it can be shown that the nonnormalized variables satisfy the following ODEs:

(3) $$\begin{array}{c} \frac{dY_{1}}{dt}=s_{1}Y_{1}-\frac{r}{\sum_{i=1}^{4}Y_{i}}\left(Y_{1}Y_{4}-Y_{2}Y_{3}\right),\\ \frac{dY_{2}}{dt}=s_{2}Y_{2}+\frac{r}{\sum_{i=1}^{4}Y_{i}}\left(Y_{1}Y_{4}-Y_{2}Y_{3}\right),\\ \frac{dY_{3}}{dt}=s_{3}Y_{3}+\frac{r}{\sum_{i=1}^{4}Y_{i}}\left(Y_{1}Y_{4}-Y_{2}Y_{3}\right),\\ \frac{dY_{4}}{dt}=-\frac{r}{\sum_{i=1}^{4}Y_{i}}\left(Y_{1}Y_{4}-Y_{2}Y_{3}\right). \end{array}$$

Note that rescaling all $Y_{i\;}$ by a constant leaves $X_{i\;}$invariant. To fix this scaling, we use $X_{i}\left(0\right)=Y_{i}\left(0\right)\;$for all *i* = 1, …, 4. As outlined in Appendix A.1, the following approximate solutions of the ODEs (3) can be found, assuming that both mutations *A* and *B* arise on background *ab* (so that $X_{1}\left(0\right)=0)$:

(4) $$\begin{array}{c} Y_{1}\left(t\right)\approx rX_{2}\left(0\right)X_{3}\left(0\right)e^{s_{1}t}\int_{0}^{t}\frac{1}{X_{4}\left(0\right)+X_{2}\left(0\right)e^{s_{2}\tau }+X_{3}\left(0\right)e^{s_{3}\tau }}d\tau ,\\ Y_{2}\left(t\right)\approx X_{2}\left(0\right)e^{s_{2}t}\left[1-rX_{3}\left(0\right)\int_{0}^{t}\frac{e^{s_{3}\tau }}{X_{4}\left(0\right)+X_{2}\left(0\right)e^{s_{2}\tau }+X_{3}\left(0\right)e^{s_{3}\tau }}d\tau \right],\\ \;Y_{3}\left(t\right)\approx X_{3}\left(0\right)e^{s_{3}t}\left[1-rX_{2}\left(0\right)\int_{0}^{t}\frac{e^{s_{2}\tau }}{X_{4}\left(0\right)+X_{2}\left(0\right)e^{s_{2}\tau }+X_{3}\left(0\right)e^{s_{3}\tau }}d\tau \right],\\ Y_{4}\left(t\right)\approx X_{4}\left(0\right)+rX_{2}\left(0\right)X_{3}\left(0\right)\int_{0}^{t}\frac{e^{s_{1}\tau }}{X_{4}\left(0\right)+X_{2}\left(0\right)e^{s_{2}\tau }+X_{3}\left(0\right)e^{s_{3}\tau }}d\tau . \end{array}$$

These approximations were first established and tested by Yun Song (personal communication). Numerical analysis suggests that they are generally excellent for $r<min\left(\;s_{2},s_{3}\right)$/10. In the following we refer to the scenario described by Equation (4) as the repulsion case. Similar equations can be obtained for the association case, for which we assume that *B* occurs on the same chromosome as *A*; i.e., $X_{1}\left(0\right)>0$ and $X_{3}\left(0\right)=0.$

## 3. Fixation Times

Using Equation (4), we can find the fixation time of two interfering mutations. We call an allele or haplotype fixed when it reaches frequency 1−δ and denote this time T. Thus, T measures the time from some initial frequency at t=0 to 1−δ. The initial frequency may be the frequency of a newly arising mutation. In a haploid population of size N, which we consider here, this initial frequency is given by 1/N. In our case, however, we are interested in the fixation of the double mutant AB whose initial frequency is $X_{1}\left(0\right)=0,\;$as AB arises during the fixation process due to recombination. Thus, the fixation time of AB is found by solving the equation

(5) $$X_{1}\left(T\right)=1-\delta \;,$$

where δ is a small number.

Next we express Equation (5) in terms of nonnormalized variables and obtain

$$X_{1}\left(T\right)={\left(1+\sum_{i=2}^{4}\frac{Y_{i}(T)}{Y_{1}(T)}\right)}^{-1}=1-\delta \;.$$

Using (4) with $X_{2}\left(0\right){>X}_{3}\left(0\right)$ and $s_{3}>s_{2}$, this equation can be approximated by

(6) $$\delta \approx \frac{Y_{2}\left(T\right)}{Y_{1}\left(T\right)}+\frac{Y_{3}\left(T\right)}{Y_{1}\left(T\right)}\;.$$

Evaluating the terms on the right-hand side of Equation (6) requires that we find useful approximations of the integrals in Equation (4) because closed formulae for these integrals are not known (see Appendix A.2). In addition to the two aforementioned assumptions, we assume that T is sufficiently large such that the second mutation is eventually dominating the first one, i.e., $X_{2}(0)e^{s_{2}T}\ll X_{3}(0)e^{s_{3}T}$, and population size is large $\left(N>10^{5}\right)\;$such that selection is strong $(Ns_{i}>100).\;$Under these assumptions we find for the integrals $I_{i}\left(T\right)$ defined in Appendix A.2:

(7) $$\begin{array}{l} I_{1}\left(T\right)\approx -\frac{ln\left(X_{2}\left(0\right)\right)}{s_{2}\left(1-X_{2}\left(0\right)\right)},\\ I_{2}\left(T\right)<\frac{T}{X_{3}\left(0\right)},\\ I_{3}(T)\approx \frac{1}{X_{2}\left(0\right)}\left[\frac{s_{3}}{s_{2}\left(s_{3}-s_{2}\right)}ln\left(X_{2}\left(0\right)\right)-\frac{1}{s_{3}-s_{2}}ln\left(X_{3}\left(0\right)\right)\right]. \end{array}$$

Inserting these formulas into Equation (6), this equation can be written in the following form:

(8) $$\delta I_{1}\left(T\right)\approx \frac{1}{X_{3}\left(0\right)e^{s_{3}T}}\left(\frac{1}{r}-X_{3}\left(0\right)I_{2}\left(T\right)\right)+\frac{1}{X_{2}\left(0\right)e^{s_{2}T}}\left(\frac{1}{r}-X_{2}\left(0\right)I_{3}\left(T\right)\right)\;.$$

We can neglect the first term on the right-hand side of Equation (8) for the following reasons: first, because we assumed that $X_{2}\left(0\right)e^{s_{2}T}\ll X_{3}\left(0\right)e^{s_{3}T},$ and second since $X_{3}\left(0\right)I_{2}\left(T\right)$ is bounded by $\frac{1}{r},\;$the term $\frac{X_{3}\left(0\right)I_{2}\left(T\right)}{X_{2}\left(0\right)e^{s_{2}T}}$ can be neglected compared to $\frac{1}{X_{2}\left(0\right)e^{s_{2}T}}\frac{1}{r}$. This leads to Equation (9):

(9) $$-\frac{\delta X_{2}(0)ln\left(X_{2}\left(0\right)\right)}{s_{2}\left(1-X_{2}\left(0\right)\right)}\approx e^{-s_{2}T}\left(\frac{1}{r}-\frac{s_{3}ln\left(X_{2}\left(0\right)\right)}{s_{2}\left(s_{3}-s_{2}\right)}+\frac{ln\left(X_{3}\left(0\right)\right)}{s_{3}-s_{2}}\right)\;.$$

Finally, we introduce population size N into this equation by writing $\delta =\frac{1}{N}{\;,\;X}_{3}\left(0\right)=\frac{1\;}{N}$ and $X_{2}\left(0\right)=\frac{x_{20}}{N}$, where $x_{20}\;$is the number of A alleles at t=0. Then solving the equation for T yields an explicit expression for the fixation time in the repulsion case:

(10) $$T\approx \frac{1}{s_{2}}\left[2ln\left(N\right)-ln\left(\frac{x_{20}}{s_{2}}\right)-ln\left(\frac{ln\left(\frac{N}{x_{20}}\right)}{1-\frac{x_{20}}{N}}\right)+ln\left(\frac{1}{r}+\frac{s_{3}}{s_{2}}\frac{ln\left(\frac{N}{x_{20}}\right)}{s_{3}-s_{2}}-\frac{ln\left(N\right)}{s_{3}-s_{2}}\right)\right]\;.$$

Table 1 shows that this result agrees very well with the numerical solution of Equation (8).

The first five columns show the simulation data: $X_{2}\left(0\right),\;$the frequency of the first mutation when the second mutation is introduced; $\pi_{l},$ relative genetic diversity at a neutral locus between loci 1 and 2 near locus 1 (here ‘relative’ refers to expected diversity under neutrality); $\pi_{m},$ relative genetic diversity at a neutral locus in the middle between loci 1 and 2; $\pi_{r},$ relative genetic diversity at a neutral locus between loci 1 and 2 near locus 2; D, Tajima’s measure of the deviation of the level of variation from neutrality at the locus in the middle. $T_{ana}$ is the fixation time from Equation (10) measured in generations, and $T_{num\;}$is obtained by solving Equation (8) numerically. The parameter values are: $N=20,000,\;X_{3}\left(0\right)=\frac{1}{N},\;\delta =\frac{1}{N},\;s_{2}=0.1,\;s_{3}=0.2,\;r=0.005.$

As expected, the formula for T is complex. However, in the interesting parameter range of small r values such that$\;\frac{1}{r}\gg \left|\frac{s_{3}}{s_{2}}\frac{ln\left(\frac{N}{x_{20}}\right)}{s_{3}-s_{2}}-\frac{ln\left(N\right)}{s_{3}-s_{2}}\right|$, we may approximate Equation (10) as

(11) $$T\approx \frac{1}{s_{2}}\left[2ln\left(N\right)-ln\left(\frac{r}{s_{2}}\frac{x_{20}}{1-\frac{x_{20}}{N}}ln\left(\frac{N}{x_{20}}\right)\right)\right]$$

This approximation works very well for small introduction frequencies $X_{2}\left(0\right)$, i.e., $X_{2}\left(0\right)=0.007$ and 0.024 for the parameter values given in Table 1. The first term on the right-hand side of Equation (11) equals the fixation time of a new allele starting at frequency $\frac{1}{N\;}\;$and ending at$\;1-\frac{1}{N\;}$, driven by positive directional selection with selection coefficient$\;s_{2}$. This term also appears in the result of Bossert and Pfaffelhuber [7]. The denominator $s_{2}\;$can be explained as follows. To go to fixation, the successful recombinant *AB* with fitness $1+s_{2}+s_{3\;}\;$has to compete against the—at the time—dominant *aB* type with fitness $1+s_{3\;},\;$having a fitness advantage $s_{2}.$

Furthermore, unless r is very small, the second term in Equation (11) is negative such that T is smaller than $\frac{2ln\left(N\right)}{s_{2}}$. This is not surprising, as we are dealing here with an equation describing continuous input of new AB alleles due to recombination, similar to the case of fixation under continuous mutation pressure and positive directional selection ([16], Equation (8)). Thus, based on Equation (11), we obtain a relatively simple formula for the threshold of r

(12) $$r_{c}=\frac{s_{2}}{x_{20}}\frac{1-\frac{x_{20}}{N}}{ln\left(\frac{N}{x_{20}}\right)},$$

above which recombination speeds up fixation time. For $r<r_{c}$, however, the second term in Equation (11) turns positive, such that the fixation time T becomes larger than $\frac{2ln\left(N\right)}{s_{2}}\;$, meaning that the input of recombinants ceases.

In the association case the fixation time can be derived in a similar way. However, an explicit expression for the fixation time as in Equation (10) is not possible. Instead, we end up in a transcendental equation that can in general only be solved numerically.

## 4. Genomic Footprint of Competing Mutations

In this section, we analyze simulation data from a study of genetic variation at neutral sites located between two selected loci [12]. The data were obtained using Monte Carlo simulations of a Wright-Fisher model ([13], chapt. 3) with two selected loci and three neutral loci. The three neutral sites are located between the selected loci as described in Table 1. Table 1 also contains the parameter values used in the simulations. They meet the assumptions of our analysis, except for the population size. In the simulations N=20,000 was used, while in our derivation $N>10^{5}\;$was suggested. To check whether this causes problems, we compared the analytical results for *T* from Equation (10) with the numerical solutions of Equation (8) for N=20,000. However, as Table 1 (columns 6 and 7) shows, no discrepancies could be found.

The first observation concerns *T* as a function of $X_{2}(0)$. Since r=0.005 was used in all simulations and in all cases r is larger than the threshold $r_{c}$ (Equation (12)), we expect that *T* is smaller than $\frac{2ln\left(N\right)}{s_{2}}=198.1\;$and decreases with increasing $X_{2}\left(0\right)=\frac{x_{20}}{N}$. This is indeed the case. The most pronounced effect of $X_{2}\left(0\right)\;$on fixation time is observed for small values of $X_{2}\left(0\right)<0.1.$ For larger values of $X_{2}\left(0\right)$, however, fixation time is relatively constant. This observation is consistent with the formulas for *T*, especially Equation (11), which shows that, for given$\;\frac{r}{s_{2}}$, fixation time depends logarithmically on $x_{20}$. This formula also says that recombination is most important in speeding up fixation when the second mutation is introduced at low $X_{2}\left(0\right)\;$values. Here the interference between the two mutations is largest.

Next we discuss the simulation results of Chevin et al. [12] in light of our analysis. Since these simulations assume that the introduction of the second mutation into the population occurs in repulsion or association with *A*, depending on the frequency of *A*, we averaged the times to fixation for both cases. Average fixation time exhibits the same features as the fixation time for the repulsion case alone: a steep decay of the fixation time for small $X_{2}\left(0\right)\;$and a rather constant level for larger introduction frequencies. This is because the fixation times in the association case are substantially shorter than in the repulsion scenario and show a narrow range from 104.8 generations for $X_{2}\left(0\right)=0.007\;$to 97.0 generations for $X_{2}\left(0\right)=0.5.$ This shows very clearly that the fixation time is largely determined by the repulsion phase.

Regarding variation at the neutral loci, Chevin et al. report that the two loci close to the selected ones show typical hitchhiking effects [8]; i.e., variation is reduced relative to the neutral standard level such that stronger selection acting at locus 2 $\left(s_{3}>s_{2}\right)\;$leads to a greater reduction than at the neutral site near locus 1. Furthermore, variation at the neutral locus in the middle between the two selected loci is greater than that expected for selection at a single locus with selection coefficient $s_{2}\;$or $s_{3}$. Such an excess of neutral variation between two selected sites was also observed by Kim and Stephan [17]. It is essential for our analysis and will be further discussed below.

Increasing $X_{2}\left(0\right)\;$generally leads to stronger hitchhiking effects such that levels of neutral variation decrease with $X_{2}\left(0\right)$. This can be clearly observed at the neutral locus close to locus 1, whereas at the neutral locus close to the stronger selected site, this is hardly visible. At the neutral locus in the middle there is also a strong decay of variation with increasing levels of $X_{2}\left(0\right)$. The effect of $X_{2}\left(0\right)$ on hitchhiking is likely due to the interference of the two mutations. The longer they compete with each other on their way to fixation, the weaker their hitchhiking effect. This has already been observed in other studies (e.g., [17]).

Finally, we discuss *D*, a statistic introduced by Tajima [18]. In Table 1 (column 5) only the *D* values for the neutral locus in the middle between locus 1 and 2 are shown. All *D* values at the other two loci are negative as expected from the theory of genetic hitchhiking. A negative *D* is observed when an allele has either a lower or higher frequency than expected by the neutral theory. Interestingly, however, Chevin et al. [12] observed strongly positive *D* values for $X_{2}\left(0\right)=0.007,\;0.024,\;$and 0.077, whereas *D* is around zero or negative for larger $X_{2}\left(0\right).$ Positive values of *D* indicate that alleles are at intermediate frequencies, such as predicted for balancing selection. In our case, however, this is probably not a valid hypothesis, at least concerning the standard models of balancing selection. A plausible hypothesis proposed by Bossert and Pfaffelhuber [7] is that positive *D* may be observed when a haplotype structure arises in the genome through recombination between different haplotypes consisting of multiple polymorphic loci. Haplotype structures exist in populations only if polymorphisms at individual loci tend to be in intermediate frequency (such that the less frequent variants are not too rare). This may be the case for $X_{2}\left(0\right)=0.007,\;0.024,\;$and 0.077, but not for the larger $X_{2}\left(0\right)$ values, for which diversity is more heavily reduced (Table 1). An alternative, though related, hypothesis postulates that the dynamics of the two selected mutations (while in repulsion) reach nonnegligible frequencies at similar times such that recombination may produce haplotypes with the two favorable alleles in coupling [12].

If these hypotheses are correct, a genomic footprint of competing beneficial mutations may be detected by measuring Tajima’s *D* and/or linkage disequilibrium. In general, footprints associated with selective sweeps caused by the fixation of beneficial mutations can be found in genetic data if their characteristic pattern of variation, such as a dip of nucleotide diversity around a selected site or a haplotype structure revealed by linkage disequilibrium, persists for some time. For Wright-Fisher populations, such signatures may be detected for up to 0.1N generations after fixation of the driving mutations [19,20].

## 5. Discussion

For a highly beneficial mutation *A* at locus 1 spreading in a very large population, we have analyzed the scenario when a second beneficial mutant *B* arises before *A* has fixed. Under the assumptions that the fitness of *B* is greater than that of *A* and that *A-* and *B*-carrying chromosomes can recombine, recombinants *AB* may form and eventually fix. We present approximate formulas for the fixation time of *AB* under additive fitness of the mutations as a function of $X_{2}\left(0\right),\;$the frequency of *A* at the introduction of *B*. The latter parameter turns out to be useful for describing the interference between competing beneficial mutations.

Our analysis suggests that the effect of interference between beneficial mutations is most pronounced for small values of $X_{2}\left(0\right)<0.1.\;$In this parameter range, fixation time decreases substantially with $X_{2}\left(0\right).$ However, for larger values, fixation time is relatively constant (Table 1). This agrees with the formulas for *T*, especially Equation (11), which shows that *T* depends logarithmically on $X_{2}\left(0\right)$. We also observed that fixation time is largely determined by the conditions of the repulsion case.

Similarly, the effect of interference on the genomic footprint of competing mutations can be clearly discerned. For small values of $X_{2}\left(0\right)=0.007,\;0.024,\;$and 0.077, a strongly positive *D* was observed, whereas *D* is around zero or negative for larger $X_{2}\left(0\right)$ (Table 1). Positive values of *D* indicate that alleles are in intermediate frequencies. This may be observed when a haplotype structure arises in the genome through recombination between different allelic types consisting of multiple polymorphic loci [7,12].

Finally, we address the question of whether we can expect to observe patterns of overlapping selective sweeps due to competing mutations in recombining genomic regions. Estimates of the average selection coefficient s and the rate ν at which beneficial mutations arise and go to fixation (i.e., selective substitutions) are known for some species, including *Drosophila melanogaster*. For instance, Jensen et al. [21] analyzed a dataset of genetic variation from the euchromatic part of the genome of a *D. melanogaster* population from Africa, which is—roughly speaking—the recombining portion of chromosomes. They obtained the following estimates: s=0.002, $N=5\times 10^{6}\;$and $\nu =4.2\;{\times 10}^{-11}\;$per generation per nucleotide site. Since under selection and genetic drift the mean fixation time (conditional on fixation) for a diploid species such as *D. melanogaster* is $T=\frac{4}{s}ln\left(2Ns\right)$ [3], we find that the probability of a second substitution arising on a chromosome during the sojourn of the first one to fixation is $T\nu =8.3\times 10^{-7}$ per nucleotide site. Multiplying Tν with the size of the euchromatic part of a chromosome (in *D. melanogaster* approximately 24 Mb $=2.4\times 10^{7}\;$base pairs), we find that on average at about 20 sites of a chromosome strongly selected substitutions could arise and compete with the first mutation during its sojourn to fixation. However, only a part of these substitutions can cause interference. According to our results, a necessary condition for interference to occur is that the second mutation arises as long as the first mutation is at low frequency, say $X_{2}\left(0\right)<0.1.\;$The sojourn time of the first mutation (with selection coefficient s) in an interval $\left[\frac{1}{N},\;X_{2}\left(0\right)\right]\;with\;X_{2}\left(0\right)=0.1$ is approximately $ln\left(2NX_{2}\left(0\right)\right)$ generations, which includes drift (calculated from Equations (4.25) and (4.41) in [13]). These results suggest that beneficial mutations spend a relatively long time (in this case about 35% of their fixation time) at low frequencies (≤0.1) before they go on to fixation. In the *Drosophila* case, about 7 of the 20 selected mutations may therefore cause interference. This number, however, could be even higher if—in addition to genetic drift—other diversity-reducing forces such as background selection are incorporated [22].

An example that some of these selected substitutions occur in close proximity in a recombining region of the genome is found at the *polyhomeotic* locus of a European population of *D. melanogaster*. Voigt et al. [23] report a case in which five selected substitutions (i.e., nearly fixed variants between Europe and Africa) are located in the 5-kb intergenic region between *polyhomeotic proximal* and the gene *CG3835* within a segment of 2.28 kb. They showed that these five selected variants are involved in the adaptation of *D. melanogaster* to the higher temperature in Europe compared to that of the ancestral species range in Africa. Variation is generally low in the whole *polyhomeotic* region, and Tajima’s *D* is strongly negative, as expected after a sweep. However, using a larger dataset than in her previous study, Susanne Voigt (personal communication) found evidence that the five beneficial substitutions that likely caused the sweep did not act independently in a sequential manner but were selected as a haplotype block. As a consequence, an elevated level of Tajima’s *D* in the fragment containing the five selected substitutions was not detected.

A more promising example in the context of interference between beneficial mutations may be the *Agouti* locus in deer mice. Here the precise mutations required for adaptation to the light-colored soil of the Nebraska Sand Hills have been identified [24]. The authors claim that—contrary to the aforementioned *Drosophila* case—the light Sand Hills phenotype is the result of independent selection on many mutations within the *Agouti* locus spanning about 120 kb. Thus, in this case, a genomic footprint of interference between beneficial mutations may be encountered in sequence data.

## 6. Conclusions

For a highly beneficial mutation *A* spreading in a very large population, we have analyzed the scenario that at a closely linked locus a second beneficial mutant *B* arises before *A* fixes. Under the assumptions that the fitness of *B* is greater than that of *A* and that *A-* and *B*-carrying chromosomes can recombine, recombinants *AB* may form and eventually fix. We presented explicit formulas for the fixation time of *AB* as a function of $X_{2}\left(0\right),\;$which denotes the frequency of *A* at the time when *B* arises. Our analysis suggests that the effect of interference between the beneficial mutations is most pronounced for small values of $X_{2}\left(0\right)<0.1.$ Furthermore, we identified a threshold value for r, below which fixation of *AB* slows down. We linked our results to published simulation data that describe the genomic footprint of interfering selective sweeps. We find that—as expected—in parallel to fixation time, the postulated excess of intermediate-frequency variants at neutral sites between the two linked selected loci occurs when interference is strong; i.e., $X_{2}\left(0\right)<0.1$. Finally, we addressed the question under which circumstances interference between selective sweeps may be observed in recombining genomic regions of a chromosome. We conclude that our results are important for the rapidly growing field of population genomics in interpreting DNA sequence data from chromosome-wide studies, in which simultaneously occurring selective sweeps are frequent.

## Figures and Tables

**Table 1:** Simulation data of Chevin et al. [12] and fixation times.

$X_{2}(0)$	$\pi_{l}$	$\pi_{m}$	$\pi_{r}$	*D*	$T_{ana}$	$T_{num}$
0.007	0.333	0.491	0.191	0.503	162.7	163.4
0.024	0.311	0.473	0.201	0.535	157.0	152.1
0.077	0.276	0.420	0.198	0.468	141.8	141.8
0.224	0.244	0.323	0.192	0.019	133.1	133.2
0.5	0.184	0.184	0.168	−0.621	127.1	127.3

## Data Availability

The data analyzed are contained in Table 1.

## Appendix A

### Appendix A.1. Derivation of the Approximate Solutions of ODEs (3)

We write the solutions in the form

(A1) $$\begin{array}{l} Y_{1}=rX_{2}\left(0\right)X_{3}\left(0\right)e^{s_{1}t}I_{1}\left(t\right),\\ Y_{2}=X_{2}\left(0\right)e^{s_{2}t}\left(1-rX_{3}(0)I_{2}\left(t\right)\right),\\ Y_{3}=X_{3}\left(0\right)e^{s_{3}t}\left(1-rX_{2}(0)I_{3}\left(t\right)\right),\\ Y_{4}=X_{4}\left(0\right)+rX_{2}\left(0\right)X_{3}\left(0\right)I_{4}\left(t\right), \end{array}$$

where $I_{i}\left(0\right)=0$ for *i* = 1, …, 4.

Next we compare the time derivatives of Equation (A1) with those of the corresponding Equation (3). Assuming *r* << *s_i_* then leads to

(A2) $$\frac{{dI}_{4}}{dt}\approx \frac{e^{s_{1}t}}{X_{4}\left(0\right)+X_{2}\left(0\right)e^{s_{2}t}+X_{3}\left(0\right)e^{s_{3}t}}.$$

This immediately yields the last Equation in (4) (up to first order in *r*). In a similar way, we obtain $\frac{{dI}_{i}}{dt}$ for *i* = 1, 2, 3 by comparing the time derivatives of Equation (A1) with those of the corresponding Equation (3).

### Appendix A.2. Approximations of the Integrals in Equation (4)

We begin by approximating the integral

(A3) $$I_{1}\left(t\right)=\int_{0}^{t}\frac{1}{X_{4}\left(0\right)+X_{2}\left(0\right)e^{s_{2}\tau }+X_{3}\left(0\right)e^{s_{3}\tau }}d\tau \;.$$

The function to integrate has a maximum at τ=0 and decays quickly to zero (within about $\hat{t}\;\approx -\frac{ln\left(X_{2}\left(0\right)\right)}{s_{2}}\;$generations). Because $X_{3}\left(0\right)=\frac{1}{N},\;$we may assume for large populations that within this short decay time $X_{3}\left(0\right)e^{s_{3}\tau }\ll X_{2}\left(0\right)e^{s_{2}\tau }$and neglect the last term in the denominator. For t> $\hat{t}\;$the integral can then be approximated by a standard formula. This leads to the approximation for $I_{1}\left(T\right)\;$given in Equation (7). This approximation is excellent for large population sizes $N>10^{5}.$

Next we consider the integral

(A4) $$I_{2}\left(t\right)=\int_{0}^{t}\frac{e^{s_{3}\tau }}{X_{4}\left(0\right)+X_{2}\left(0\right)e^{s_{2}\tau }+X_{3}\left(0\right)e^{s_{3}\tau }}d\tau .$$

Here the function to integrate increases monotonically up to a level of $\frac{1}{X_{3}\left(0\right)}\;$for large t. This leads to the upper bound of this integral given in Equation (7). A more precise approximation is not required in this case (see main text below Equation (8)).

Finally, we approximate the integral

(A5) $$I_{3}\left(t\right)=\int_{0}^{t}\frac{e^{s_{2}\tau }}{X_{4}\left(0\right)+X_{2}\left(0\right)e^{s_{2}\tau }+X_{3}\left(0\right)e^{s_{3}\tau }}d\tau .$$

The function to integrate has a maximum at $\hat{t}=\frac{1}{s_{3}}ln\left(\frac{s_{2}}{s_{3}-s_{2}}\frac{X_{4}\left(0\right)}{X_{3}\left(0\right)}\right)\approx -\frac{1}{s_{3}}ln\left(X_{3}\left(0\right)\right).$ For $t\leq \hat{t}$ we integrate the function $\frac{e^{s_{2}\tau }}{X_{4}\left(0\right)+X_{2}\left(0\right)e^{s_{2}\tau }}$ and for larger times $\frac{1}{X_{2}\left(0\right)+X_{3}\left(0\right)e^{\left(s_{3}-s_{2}\right)\tau }}$. This leads to the third formula in Equation (7), which was obtained assuming large populations.

In general, the analytical results in this paper were derived for large populations ($N>10^{5}).$ In the applying these results to the simulation data of Chevin et al. [12], we had to check numerically whether they are still valid for a smaller population size of 20,000 used in these simulations. We found that the fixation times analytically calculated agree very well with the numerical results (see Table 1).
